# Isoalantolactone inhibits pancreatic ductal adenocarcinoma progression via direct targeting of NLRP3-mediated inflammation-angiogenesis axis

**DOI:** 10.3389/fphar.2026.1840118

**Published:** 2026-07-15

**Authors:** Ying Zhang, Changquan Liu, Shengjiang Liu, Yanchun Peng, Jigang Pan, Yingnan Song, Hongjin Chen

**Affiliations:** 1 Center for Tissue Engineering and Stem Cell Research, Translation Medicine Research Center, Guizhou Biomanufacturing Laboratory, Guizhou Medical University, Guiyang, China; 2 Department of Pharmacology, School of Basic Medical Sciences, Guizhou Medical University, GuiAn, Guizhou, China; 3 Hepatobiliary and Pancreatic Surgery, Xingyi People’s Hospital, Xingyi Hospital Affiliated to Guizhou Medical University, Xingyi, Guizhou, China; 4 Department of Physiology & Pathophysiology, School of Basic Medical Sciences, Guizhou Medical University, GuiAn, Guizhou, China

**Keywords:** Angiogenesis, inflammatory, isoalantolactone, NLRP3, pancreatic ductal adenocarcinoma

## Abstract

**Background:**

Pancreatic ductal adenocarcinoma (PDAC) is the fifth most common malignancy globally, with tumor uncontrolled angiogenesis being major causes of therapeutic failure and patient death. Isoalantolactone (IATL), a natural compound, exhibits antioxidant, anti-inflammatory, anti-proliferative, and anti-tumor properties. However, its role in inhibiting tumor angiogenesis in pancreatic cancer and the underlying mechanisms remain unclear. This study aims to investigate the anti-angiogenic effects of IATL in PDAC and elucidate the associated molecular pathways.

**Methods:**

*In vitro* experiments were performed using PDAC cell lines (Panc02, PANC-1, and SW 1990) and HUVECs. *In vivo* studies were conducted using an orthotopic pancreatic cancer model in C57BL/6 mice. Cell viability, wound-healing, tube formation, *in vivo* imaging system analysis, laser speckle contrast imaging, immunohistochemistry, immunofluorescence, RT-qPCR, ELISA, and Western blot assays were used to evaluate the effects of IATL on tumor growth, angiogenesis, and inflammatory responses. Molecular docking and cellular thermal shift assay were performed to assess the interaction between IATL and NLRP3.

**Results:**

IATL significantly inhibited the proliferation and migration of Panc02, PANC-1, and SW1990 cells. In the orthotopic pancreatic cancer model, IATL dose-dependently suppressed tumor growth, as evidenced by reduced IVIS fluorescence signals and tumor volume. IATL also markedly inhibited angiogenesis, as shown by reduced HUVECs migration and tube formation, decreased tumor blood perfusion detected by laser speckle imaging, and downregulated CD34 and VEGFA expression both *in vivo* and *in vitro*. Network pharmacology, molecular docking, and cellular thermal shift assay identified NLRP3 as a direct target of IATL. Mechanistically, IATL suppressed NLRP3 inflammasome activation, reduced ASC speck formation, inhibited the NLRP3/IL-1β signaling axis, and decreased the expression of inflammatory cytokines, including IL-6, TNF-α, IL-1β, and IL-18. NLRP3 knockdown mimicked the effects of IATL, whereas NLRP3 overexpression partially reversed its anti-tumor, anti-angiogenic, and anti-inflammatory effects, further supporting the target specificity of IATL.

**Conclusion:**

IATL functions as a novel NLRP3 pathway inhibitor, suppressing angiogenesis through anti-inflammatory mechanisms, thereby effectively inhibiting PDAC progression. These findings suggest that IATL holds potential as a therapeutic agent for pancreatic cancer.

## Graphical Abstract

**Figure:**
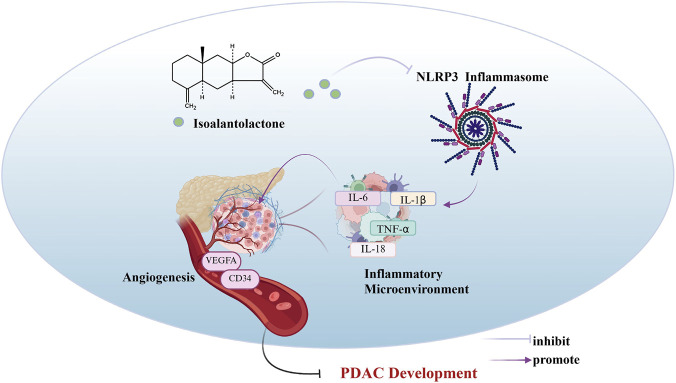

## 1 Introduction

Pancreatic ductal adenocarcinoma (PDAC) is a highly malignant tumor in the digestive system, often referred to as the “king of cancers.” According to the 2025 Cancer Data, the incidence and mortality rates of pancreatic cancer are showing a continuous upward trend worldwide, with a 5-year survival rate of only 13%. It has now become the third leading cause of cancer-related deaths in the United States (Siegel et al., 2025). The pathogenesis of PDAC is highly complex, involving interactions between genetic and environmental factors. For example, an individual’s susceptibility to PDAC may be increased by risk factors such as family history, chronic pancreatitis, diabetes, obesity, smoking, and alcohol consumption (Jacobs and Stoffel, 2024). However, due to the absence of early symptoms and the lack of sensitive biomarkers for early diagnosis, most patients are diagnosed at an advanced stage, resulting in poor treatment outcomes (Fogli et al., 2019; Zhao et al., 2025). Given the complexity of PDAC pathogenesis and the limited efficacy of current therapeutic options, identifying novel treatment strategies and developing more effective drugs have become critical research priorities.

Angiogenesis is the process of forming new blood vessels from pre-existing ones and it plays a critical role in various physiological and pathological conditions (Dudley and Griffioen, 2023a). The abnormal activation of angiogenesis is closely related to the occurrence and development of many major diseases in human, such as diabetic retinopathy, rheumatoid arthritis, vascular formation in atherosclerotic plaque and tumor (Dudley and Griffioen, 2023b). In PDAC, abnormal angiogenesis is a key contributor to malignant proliferation and metastasis (Huang et al., 2023). A 2011 Nature study reported that aberrantly activated angiogenesis is not only a key driver of tumor invasion and metastasis, but also a critical factor leading to chemotherapy resistance (Carmeliet and Jain, 2011). In tumor tissue, newly formed blood vessels provide essential nutrients for tumor growth and act as “highways” for tumor cell dissemination, thereby facilitating the metastasis of cancer cells from the primary site (Carmeliet and Jain, 2011). In PDAC, various angiogenic factors are overexpressed, including key molecules such as vascular endothelial growth factor (VEGF) and hypoxia-inducible factor-1α (HIF-1α), which maintain tumor cell survival and impair the efficacy of chemotherapeutic agents (Kim et al., 2022). Additionally, endothelial-like cancer-associated fibroblasts in PDAC promote angiogenesis and facilitate distant metastasis of tumor cells by forming angiogenic mimics and secreting pro-oncogenic factors such as VEGFA (Sun et al., 2025). Furthermore, chronic inflammation is a well-known inducer of angiogenesis and significantly contributes to vascular formation in PDAC (Matsuo et al., 2009; Imafuji et al., 2019). For example, during chronic inflammation, a variety of cytokines and chemical mediators such as tumor necrosis factor-α (TNF-α), platelet-derived growth factor (PDGF), and vascular endothelial growth factor (VEGF) are released, promoting endothelial cell proliferation and angiogenesis (Chen et al., 2025; Birtolo et al., 2016; Poh and Ernst, 2023). In addition, these factors are known to regulate neovascularization through specific signaling proteins, including the VEGF receptor (VEGFR) and CD34 (Lugano et al., 2020), while other studies have shown that chronic inflammation also promotes neovascularization via such proteins as VEGFR and CD34, thereby accelerating tumor cell metastasis (Fu et al., 2023; Sadikan et al., 2023; Zalewski et al., 2023). Consequently, therapeutic strategies targeting inflammation to suppress angiogenesis may offer novel approaches for PDAC management.

Isoalantolactone (IATL) is a sesquiterpene lactone derived from Inula helenium L., and has been a historically significant component of traditional Chinese medicine. It has been demonstrated to have potent anti-inflammatory properties, and emerging evidence suggests that it also exerts anti-cancer effects by modulating inflammatory signaling pathways. For example, IATL inhibits IKKβ kinase activity, thereby suppressing NF-κB/COX-2 signaling-driven tumorigenesis in glioblastoma (Wan et al., 2017), while in breast cancer, IATL downregulates the expression of angiogenesis-related factors such as MMP-9 and VEGF, ultimately impairing tumor angiogenesis (Jaikaria et al., 2025). However, the molecular mechanisms underpinning the anti-pancreatic cancer effects of IATL remain incompletely elucidated. In this study, we employed network pharmacology, in addition to *in vitro* and *in vivo* experiments, to investigate the effects of IATL on PDAC cell proliferation, migration, invasion, and apoptosis, as well as its influence on the inflammatory tumor microenvironment (TME) and angiogenesis-related signaling pathways. These findings provide both theoretical and experimental support for the potential of IATL as a therapeutic agent for PDAC.

## 2 Materials and methods

### 2.1 Reagents and antibodies

Isoalantolactone (IATL, Catalog No.I350991) was purchased from Aladdin (China). IATL was dissolved in cell-grade DMSO and stored at −20 °C. ATP (Catalog No.HY-B2176) and Nigericin (Catalog No.HY-127019) The primary antibody against NLRP3 (Catalog No. DF7438) was obtained from Affinity (China). Primary antibodies against VEGFA (Catalog No. A0280), IL-1β (Catalog No. A16288), IL-18 (Catalog No. A1115), ASC (CatalogNo.A1170SP)and CD34 (Catalog No. A19015) were purchased from ABclonal (China); anti-GAPDH (Catalog No.20536), GSDMD (Catalog No.20770-1-AP) and caspase (Catalog No.81482-1-RR) were sourced from proteintech (China). The goat anti-mouse (Catalog No. AS003) and anti-rabbit (Catalog No. AS014) IgG secondary antibodies were supplied by ABclonal (China).

### 2.2 Cell culture

PDAC cell lines, including Panc02, PANC-1, and SW 1990, were acquired from the Institute of Biochemistry and Cell Biology, Shanghai (Shanghai, China), and human umbilical vein endothelial cells (HUVECs) were kindly provided by Wenzhou Medical University. All cells were cultured in a complete DMEM (Gibco, CA, United States) containing 10% FBS (Gibco, CA, United States), 100 μg/mL penicillin, and 100 μg/mL streptomycin. Cells were maintained in a humidified incubator at 37 °C with 5% CO_2_. Cells in the logarithmic growth phase were then digested and passaged with 0.25% trypsin–EDTA solution.

### 2.3 MTT cell viability assay

An MTT assay was used to evaluate the cytotoxic effects of IATL on Panc02, PANC-1, and SW1990 cells. Cells were seeded into 96-well plates at a density of 5 × 10^3^ cells per well. IATL (in DMSO) was diluted in DMEM to the final concentrations of 0, 2.5, 5, 10, 20, 40, 80, and 160 μM. Following a 24 h treatment, the cells were incubated with 10 μL of MTT (Solarbio, 922NO52, 5 mg/mL) reagent in the dark for 4 h. The culture medium was then replaced with 100 μL of DMSO to dissolve the formazan crystals, and the absorbance of the solution was measured at 490 nm using a multi-functional fluorescence microplate reader (Synergy H1).

### 2.4 Detection of cell kinetics

Cells were seeded into 96-well plates at a density of 5 × 10^3^ cells per well. After attachment, cells were treated with a specific concentration of IATL and then transferred to a Cytation 7 live-cell imaging system (BioTek Cytation 7) for the detection of kinetic changes in real-time over 24 h and 72 h.

### 2.5 Wound healing assay

Panc02, PANC-1, SW1990 and HUVECs cells were seeded into 96-well plates at a density of 5 × 10^4^ cells per well and cultured until reaching full confluence. A wound was generated in the cell layer using an automatic scratch device, after which the cells were washed with sterile PBS and then cultured in a medium containing 2% fetal bovine serum. Following treatment with different doses of IATL (0, 5, 10, 20, and 40 μM), the cells were transferred to a Cytation 7 live-cell imaging system. Images were captured every 12 h for a total of 48 h and the wound surface area was quantified using ImageJ software, from which the wound healing rate was calculated.

### 2.6 Western blot analysis (WB)

PANC-1 and SW1990 cells and tumor tissues were collected, homogenized, and lysed on ice using NP40 lysis buffer (AR0107, Boster Biological Technology) supplemented with protease and phosphatase inhibitors. Protein concentrations in the lysate were quantified using a protein assay kit (20201ES90, Yeasen Biotechnology, China). Protein samples were then separated by sodium dodecyl sulfate–polyacrylamide gel electrophoresis (SDS-PAGE) and transferred onto PVDF membranes. Mmembranes were blocked with 5% non-fat milk or bovine serum albumin (BSA) at room temperature for 2 h, followed by overnight incubation with primary antibodies at 4 °C. Next, they were incubated with the specified peroxidase-conjugated secondary antibodies at room temperature for 1.5 h. Protein bands were visualized using an ECL kit (abs920, absin), and quantitative analysis of the visualized protein bands was completed using ImageJ software. Antibodies were diluted as indicated in Supplementary Table S1.

### 2.7 RNA isolation and quantitative real-time PCR (qRT-PCR)

Total RNA was extracted from cells or tissues using RNAzol® RT RNA Isolation Reagent (RN190), RNA concentrations were measured with a ThermoNDONEC spectrophotometer (Gene Company Limited), and RNA integrity was determined by agarose gel electrophoresis. Next, cDNA was synthesized from 800 ng of total RNA using the HiScript III RT SuperMix for qRT-PCR kit (Vazyme, China). Candidate gene expression was measured using the CFX Maestro Software Version 2.3 system (Bio-Rad C1000 Touch TM Thermal Cycler, Inc.). The corresponding CT values were recorded, and the relative mRNA expression levels were quantified using the 2^−ΔΔCT^ method and normalized to 18 S rRNA, with primer sequences listed in Supplementary Table S2.

### 2.8 ELISA measurement

The ELISA kit was used to detect the cytokines IL-6 and IL-1β in the culture supernatant of PANC-1 cells. Blank wells, standard wells, and sample wells were prepared in accordance with the manufacturer’s instructions. Necessary reagents were added, and plastic wrap was used to cover the wells to prevent evaporation during the incubation process. After the final step and the addition of the stop solution, the absorbance at 450 nm was measured.

### 2.9 Cell translocation experiment (CETSA)

PANC-1 cells were seeded at 70% density in a 6-well plate. After adhesion, IATL was added for 24 h, followed by cell collection and resuspension in PBS. The supernatant was removed by centrifugation at 1000 g for 5 min. Protease inhibitors were added to PBS, and cells were resuspended. Samples were divided into seven temperature points (40,45,50,55,60,65,70 °C), heated in a metal bath for 5 min, and then cooled at 4 °C. Subsequently, the samples were lysed by repeated freeze-thaw cycles in liquid nitrogen three times. At 12,000 rpm, centrifugation was performed at 4 °C for 15 min, and the supernatant was collected. One-fourth of the 5× loading buffer was added, and the sample was boiled at 100 °C for 10 min. **Western blot** analysis was conducted to detect experimental results.

### 2.10 Cell transfection

siRNA targeting NLRP3 was biosynthesized by Changsha Banma (Biotechnology Co., Ltd.), and was used for NLRP3 knockdown; the sequences are shown in Supplementary Table S3. All plasmids were kindly provided by Wenzhou Medical University. When the cell confluence reached 70%–80%, the aforementioned siRNA and plasmids were transfected using the Reagent in accordance with the manufacturer’s protocol. 24 h later, the cells were treated with IATL (0 μM, 10 μM, 20 μM, 40 μM) for another 24 h, and the transfection efficiency was evaluated by Western blot.

### 2.11 Tube formation assay

Twenty microliters of Ceturegel® Matrix for Organoid Culture, Phenol Red-Free, LDEV-Free (Yeasen, 40191ES08) was pipetted into pre-chilled 24-well plates and spread evenly by overnight leveling at 4 °C. The next day, the plates were incubated at 37 °C for 30 min to allow the gel to solidify. Subsequently, 1.5 × 10^5^ HUVECs per well were resuspended in conditioned medium (collected from the culture medium of PANC-1 and SW1990 cells after drug treatment) and added to the 24-well plates in a volume of 500 μL. After incubation at 37 °C, tube formation was observed under an inverted microscope at 2 h and 6 h, respectively. The total number of branching points was quantified using ImageJ software.

### 2.12 NLRP3 inflammasome activation in THP-1 cells

THP-1 cells were counted and seeded in 6-well plates at a density of 1 × 10^6^ cells per well, followed by overnight incubation at 37 °C. On the next day, the cells were primed with 500 ng/mL LPS for 5 h. Subsequently, the inflammasome activator (5 mM ATP and 15 μM Nigericin) was added, and the cells were further incubated for 2 h to induce NLRP3 inflammasome activation.

### 2.13 NLRP3 inflammasome activation in PANC-1 and SW1990 cells

PANC-1 and SW1990 cells were seeded in 6-well plates at a density of 1 × 10^6^ cells per well and incubated overnight at 37 °C. The following day, the cells were stimulated with 500 ng/mL LPS for 5 h, followed by treatment with IATL for 0.5 h. Thereafter, the inflammasome activator (5 mM ATP and 15 μM Nigericin) was added, and the cells were incubated for an additional 2 h. After stimulation, the culture supernatants were collected for supernatant protein extraction. The adherent cells were then lysed with cell lysis buffer, scraped, and harvested for cellular protein extraction.

### 2.14 ASC fluorescent speck assay

THP-1, PANC-1, and SW1990 cells were seeded onto confocal dishes or slides at a density of 1 × 10^5^ cells per well and incubated overnight at 37 °C. The cells were then treated according to the NLRP3 inflammasome activation protocol described above. After treatment, the samples were fixed with 4% paraformaldehyde for 30 min at room temperature in the dark, permeabilized with 0.3% Triton X-100, and blocked with 5% BSA. The samples were subsequently incubated with ASC primary antibody at 4 °C overnight, followed by incubation with the corresponding fluorescent secondary antibody for 1 h at room temperature in the dark. Finally, the samples were mounted with anti-fade mounting medium containing DAPI and observed and photographed under a laser confocal microscope.

### 2.15 Orthotopic transplantation model of pancreatic cancer cells

The sample animals were 18-20 g male C57BL/6 mice, purchased from the Animal Center of Guizhou Medical University. All animal procedures were approved by the Animal Care and Use Committee of Guizhou Medical University, Guiyang, China. The mice were housed at stable room temperature, with free access to water and standard food, and maintained on a 12:12 h light-dark cycle. To generate an orthotopic transplantation model of Panc02 cells, cells were digested with trypsin, collected, and washed with phosphate-buffered saline, before they were orthotopically injected into the mouse pancreas at a cell density of 1 × 10^7^ cells in 15 μL to induce tumorigenesis in the mice. After 7 days, the mice were randomly divided into 3 groups: vehicle control treatment, 5 mg/kg IATL treatment, and 10 mg/kg IATL treatment. In the vehicle control treatment group, 100 μL of a mixed solution of 10% DMSO and 90% corn oil was injected intraperitoneally. In the 5 mg/kg IATL treatment group, 100 μL of IATL (5 mg/kg) diluted with a mixed reagent of 10% DMSO and 90% corn oil was injected intraperitoneally. In the 10 mg/kg IATL treatment group, 100 μL of IATL (10 mg/kg) diluted with a mixed reagent of 10% DMSO and 90% corn oil was injected intraperitoneally. Treatment started on day 7 and was administered every other day. After 30 days, subsequent experiments were carried out, after which the tumors were harvested, photographed, and measured.

To evaluate the effect of Panc02 cell tumorigenesis, Panc02 cells infected with a lentivirus expressing luciferase were used. These cells were orthotopically injected into the pancreas of mice, and experimental treatments were carried out according to the groups as previously described. At the endpoint, mice were injected intraperitoneally with luciferin (150 mg/kg), and the *in vivo* imaging system was used to quantitatively assess the tumor size.

### 2.16 Laser speckle contrast imaging

To evaluate the changes in pancreatic tumor angiogenesis after IATL treatment, mice were anesthetized with tribromoethanol, followed by exposure of pancreatic tumors for scanning with a laser speckle contrast imaging device. The PIM software system was used for image processing and analysis.

### 2.17 Hematoxylin and eosin (H&E) staining and immunohistochemistry

The harvested mouse tissues were fixed in formalin overnight, dehydrated in different gradients of alcohol, and embedded in paraffin. Paraffin-embedded tissue sections were cut at a thickness of 5 μm, then subjected to standard dewaxing and rehydration. Antigen retrieval was performed using citric acid under high-temperature and high-pressure conditions. These sections were incubated with 3% hydrogen peroxide at room temperature for 10 min to block endogenous peroxidase activity, followed by blocking with 5% BSA for 1 h at room temperature. The sections were incubated with primary antibodies against IL-6 (abs135607, 1:200) and TNF-α (#11948, 1:200) overnight at 4 °C. The next day, after returning the sections to room temperature, they were incubated with secondary antibodies for 1 h. The reaction was developed using a DAB chromogenic kit (AR1027-3), and stopped once a brown color developed. Subsequently, the sections were counterstained with hematoxylin and mounted. For H&E staining, after direct rehydration, staining was each carried out using an H&E staining kit (Solarbio, G11221).

### 2.18 Immunofluorescence

Paraffin-embedded tumor section preparation and antigen retrieval were prepared as above. After naturally cooling to room temperature, the sections were blocked with 5% BSA for 1 h. The tumor sections were then incubated overnight at 4 °C with primary antibodies against VEGFA (A0280, 1:500) and CD34 (A19015, 1:200). Following this, the sections incubated with biotinylated secondary antibodies for 30 min at 37 °C or room temperature. Finally, the sections were mounted with an anti-fade mounting medium, and representative images were captured using a laser confocal microscope (Nikon DS-Fi2).

### 2.19 Network pharmacology analysis

Putative targets of IATL were retrieved from online databases, including TCMSP (https://old.tcmsp-e.com/tcmsp.php), Pharmmapper (http://www.lilab-ecust.cn/pharmmapper/results/240927015212.html/240927015212), and Genclip3 (http://cismu.net/genclip3/analysis.php).

The targets associated with pancreatic cancer (PAAD) were obtained from the GENEcards (https://www.genecards.org), OMIM (https://www.omim.org), TTD (https://db.idrblab.net/ttd/), and Genclip3 (http://cismu.net/genclip3/analysis.php) databases.

Subsequently, the overlapping targets among IATL and PAAD were identified using VENNY(https://bioinfogp.cnb.csic.es/tools/venny). The intersection of drug–disease targets was imported into the STRING database to analyze the protein–protein interaction relationships among the targets, and the data were imported into Cytoscape software for visualization and to screen the core targets.

Gene Ontology (GO) terms and Kyoto Encyclopedia of Genes and Genomes (KEGG) pathway enrichment analyses were performed using Metascape (https://www.metascape.org), with the results visualized through the bioinformatics platform (http://www.bioinformatics.com.cn).

### 2.20 Molecular docking model

The crystal structure of NLRP3 protein (PDB ID: 8wsm) was obtained from the Protein Data Bank (https://www.rcsb.org), and the compound structure of IATL (Pubchem CID: 73285) was retrieved from the TCMSP database. Subsequently, molecular docking simulations were performed using the online platform CB-DOCK2 (http://183.56.231.194:8001/cb-dock2/index.php).

### 2.21 Statistical analysis

Statistical analyses were performed using GraphPad Prism 8.0.1 (GraphPad Software Inc., CA, United States), and band intensities were quantified using ImageJ software. All data are presented as the mean ± standard deviation (SD). Comparisons between two groups were performed using the independent-samples t-test, whereas differences among multiple groups were analyzed using one-way analysis of variance (ANOVA) followed by Tukey’s *post hoc* test for multiple comparisons. A value of P < 0.05 was considered statistically significant.

## 3 Results

### 3.1 IATL exhibits significant anticancer activity in pancreatic cancer, both *in vitro* and *in vivo*

**FIGURE 1:**
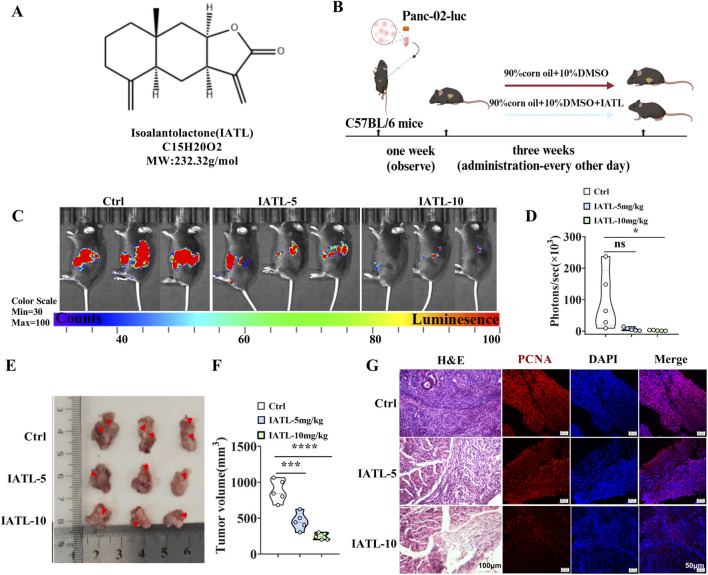
IATL exhibits significant anticancer activity in pancreatic cancer models. Mice were intraperitoneally injected with vehicle or IATL (5 or 10 mg/kg) every other day for three consecutive weeks. **(A)** Chemical structure of IATL. **(B)** Schematic overview of the *in vivo* experimental design. **(C)** Analysis of pancreatic cancer models using the *in vivo* imaging system (IVIS). **(D)** Quantification of tumor fluorescence signals in mice by IVIS. Data are presented as mean ± SD (n = 5). **(E,F)** Representative images and tumor volumes of mice treated with IATL (5 or 10 mg/kg) or vehicle control. Data are presented as mean ± SD (n = 5). **(G)** Representative H&E-stained images of pancreatic tissues from mice. Scale bar, 100 μm. Representative immunofluorescence images of PCNA (red) staining in tumor tissues from the control and IATL (5 or 10 mg/kg) treatment groups. Nuclei were counterstained with DAPI (blue). Scale bar, 50 μm (n = 3). Statistical differences were determined by one-way ANOVA, where ns indicates no significant difference, *P < 0.05, **P < 0.01, ***P < 0.001, and ****P < 0.0001.

To evaluate the anticancer activity of IATL, the chemical structure of IATL was illustrated in Figure 1A to clarify its fundamental molecular characteristics. Existing studies have demonstrated that IATL exhibits favorable pharmacological activity and antitumor potential (Zhang C. et al., 2021; Li et al., 2022). Subsequently, its antitumor efficacy was assessed in multiple pancreatic cancer cell lines, including Panc-02, PANC-1, and SW 1990. The results demonstrated that IATL exhibited significant anticancer activity (Supplementary Figure S1). Next, to validate the *in vivo* antitumor activity of IATL, a pancreatic cancer orthotopic tumor model was established in C57BL/6 mice using Panc02-luci fluorescent cells. The IATL group received intraperitoneal injections every other day at doses of 5 or 10 mg/kg (Figure 1B). The results demonstrated that after 3 weeks of IATL treatment, tumor growth in the treatment group was significantly lower than that in the control group in a dose-dependent manner (Figures 1C–F). Additionally, to further evaluate the *in vivo* therapeutic effects of IATL, H&E staining and PCNA fluorescence staining were performed (Figure 1G). The HE staining results showed that the control group exhibited tightly packed tumor cells with altered morphology, blurred boundaries, and marked nuclear atypia, characterized by large, irregular nuclei with deep staining, which may be associated with abnormal cell proliferation. These features were significantly improved in the IATL treatment group. Simultaneously, PCNA expression was significantly downregulated in the IATL treatment group, indicating that IATL inhibits tumor cell proliferation. In summary, these findings demonstrate that IATL exhibits significant antitumor effects *in vivo*, with a pronounced dose-dependent response.

### 3.2 IATL inhibits PDAC angiogenesis and impedes malignant progression of pancreatic cancer

**FIGURE 2:**
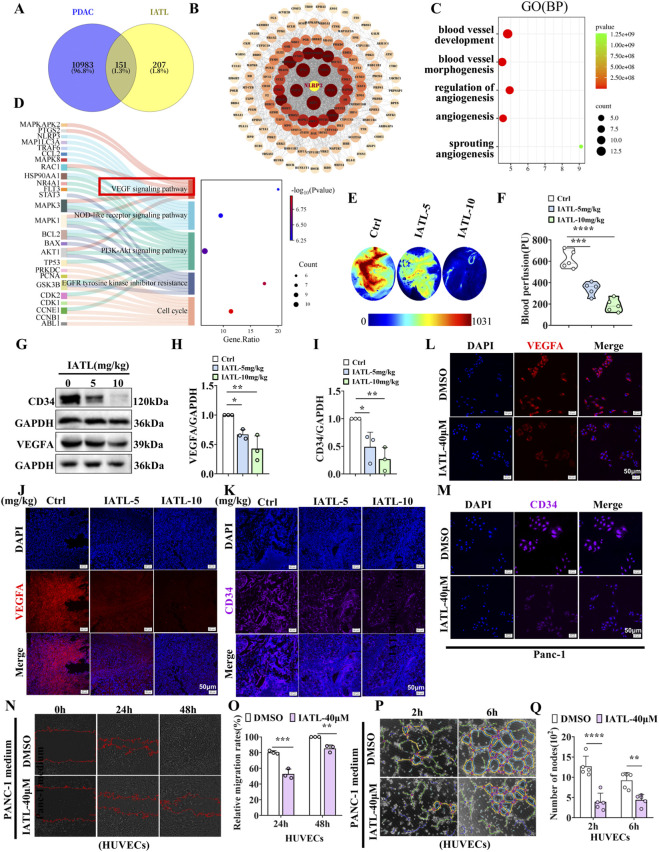
IATL demonstrates potential to inhibit PDAC angiogenesis. **(A)** Venn diagram illustrating the intersection between IATL-related targets and PDAC-related targets. **(B)** Protein–protein interaction (PPI) network of IATL targets in PDAC. **(C)** GO enrichment analysis of biological processes. IATL is involved in the angiogenesis process, with 5 enriched GO terms shown in the histogram. **(D)** KEGG pathway enrichment analysis showing that IATL participates in the classical VEGF angiogenesis pathway. The bubble plot displays 5 enriched pathways. **(E,F)** Laser speckle contrast imaging (LSCI) was used to evaluate blood perfusion in pancreatic cancer models. Data are presented as mean ± SD (n = 5). **(G–I)** Western blot analysis of CD34 and VEGFA protein levels in tumor specimens. Band intensities were quantified using ImageJ software. Data are presented as mean ± SD (n = 3). **(J,K)** Representative immunofluorescence images of CD34 (purple) and VEGFA (red) staining in tumor tissues from the control and IATL (5 or 10 mg/kg) treatment groups. Nuclei were counterstained with DAPI (blue). Scale bar, 50 μm. **(L,M)** Representative immunofluorescence images of CD34 (purple) and VEGFA (red) staining in PANC-1 cells treated with IATL (0 or 40 μM) for 24 h. Nuclei were counterstained with DAPI (blue). Scale bar, 50 μm. **(N,O)** Representative images of wound-healing assays in HUVECs co-cultured with conditioned medium. Images were acquired using the Cytation 7 live-cell imaging system. Wound area was quantified using ImageJ software, and the migration rate was expressed as a percentage of the control. Data are presented as mean ± SD (n = 3). **(P,Q)** Tube formation assays of HUVECs co-cultured with conditioned medium. Tube formation was quantified using ImageJ software. Data are presented as mean ± SD (n = 3). Statistical differences were determined by one-way ANOVA, where ns indicates no significant difference, *P < 0.05, **P < 0.01, ***P < 0.001, and ****P < 0.0001.

To investigate the potential mechanisms of IATL in treating PDAC, the potential target sites of “Isoalantolactone” were screened using databases including TCMSP, SwissTargetPrediction, PharmMapper, and Genclip3, yielding a total of 358 drug targets. Additionally, we identified 11,134 disease targets related to “pancreatic cancer” from four databases: GeneCards, TTD, OMIM, and Geneclip3. Subsequently, cross-analysis of IATL targets and PAAD targets revealed 151 potential therapeutic targets of IATL for PAAD (Figure 2A). Based on these 151 shared targets, a PPI network was constructed, comprising 135 nodes and 2,008 edges (Figure 2B). To investigate the potential mechanisms of IATL in regulating PAAD therapeutic targets, we performed gene ontology (GO) and Kyoto Encyclopedia of Genes and Genomes (KEGG) enrichment analyses. The results identified 5 significantly enriched GO terms and 5 signaling pathways (P < 0.05). The biological process (BP) terms associated with the target genes were concentrated in “angiogenesis” (Figure 2C). Furthermore, these findings suggest that IATL may regulate PDAC by participating in the VEGF angiogenesis signaling pathway (Figure 2D). Angiogenesis is one of the most critical survival modes of cancer cells, and inhibiting angiogenesis has become a hot target in anticancer therapy. First, tumor peripheral vasculature and its perfusion were evaluated using LSCI. The results showed that the tumor peripheral vascular imaging signals in mice treated with IATL were significantly lower than those in the control group, with thinner and fewer vascular images (Figures 2E,F), indicating that IATL exerts an inhibitory effect on *in vivo* angiogenesis. Subsequently, the expression of angiogenesis marker proteins was detected. Western blot results demonstrated that IATL reduced the protein levels of CD34 and VEGFA (Figures 2G–I). Further immunofluorescence staining at the cellular and animal levels demonstrated that the expression of CD34 and VEGFA was significantly reduced in the IATL-treated group compared to the control group (Figures 2J–M). To simulate the tumor microenvironment, the conditioned medium of PANC-1 cells treated with IATL was collected and used for the culture of HUVECs. Scratch assays showed that the conditioned medium significantly inhibited the migration of HUVECs in a time-dependent manner (Figures 2N,O). Subsequent *in vitro* tube formation experiments further confirmed that IATL effectively inhibited tube formation under simulated tumor microenvironment conditions (Figures 2P,Q). These findings suggest that IATL may suppress the malignant progression of pancreatic cancer by inhibiting tumor angiogenesis.

### 3.3 IATL inhibits inflammatory cytokine secretion to improve the inflammatory microenvironment of pancreatic cancer

**FIGURE 3:**
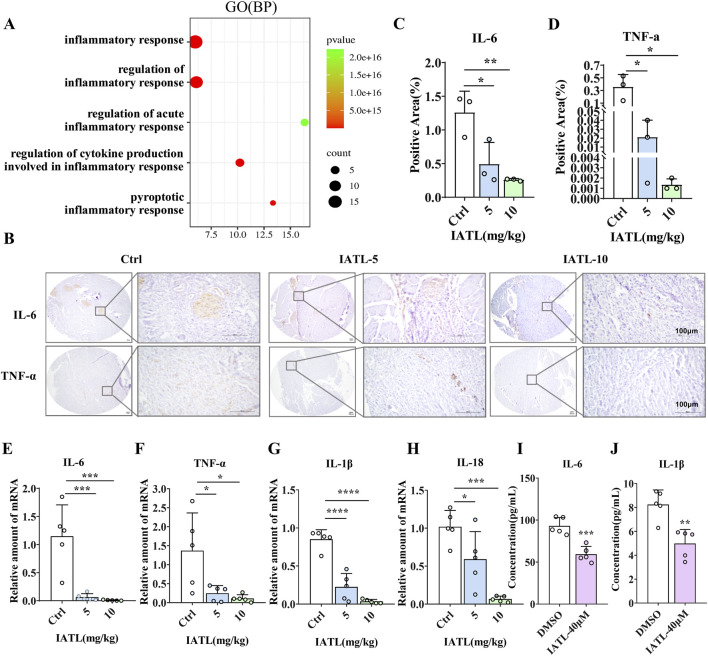
IATL inhibits inflammatory cytokine secretion and improves the inflammatory microenvironment of pancreatic cancer. **(A)** GO enrichment analysis of biological processes. IATL is involved in the inflammation process, with 5 enriched GO terms shown in the histogram. **(B)** Representative images of immunohistochemical staining for IL-6 and TNF-α in tumor tissues. Scale bar, 100 μm. **(C,D)** Quantification of brown-stained areas using ImageJ software. Data are presented as mean ± SD (n = 3). **(E–H)** RT-qPCR analysis of inflammatory cytokine mRNA levels (IL-6, TNF-α, IL-1β, and IL-18) in tumor specimens. Data are presented as mean ± SD (n = 5). **(I,J)** ELISA detection of IL-6 and IL-1β levels in the culture supernatant of PANC-1 cells treated with IATL (0 or 40 μM) for 24 h. Data are presented as mean ± SD (n = 5). Statistical differences were determined by one-way ANOVA, where ns indicates no significant difference, *P < 0.05, **P < 0.01, ***P < 0.001, and ****P < 0.0001.

Inflammatory factors in the tumor microenvironment promote the occurrence, progression, and metastasis of pancreatic cancer by mechanisms such as angiogenesis and apoptosis. Analysis of the intersecting genes revealed their significant enrichment in multiple inflammation-related biological processes (Figure 3A). Based on this, further IHC detection revealed a significant downregulation of inflammatory cytokines IL-6 and TNF-α in tumor tissues post-treatment (Figures 3B–D). Simultaneously, we detected changes in mRNA levels of inflammatory factors IL-6, TNF-α, IL-1β, and IL-18 in tumor samples, finding that IATL treatment significantly reduced the expression of these inflammatory factors (Figures 3E–H). Additionally, ELISA assays were performed to detect the expression of inflammatory cytokines IL-6 and IL-1β in the culture supernatant of PANC-1 cells treated with IATL (0,40 μM) for 24 h. Compared with the control group, IATL treatment significantly reduced the secretion of these inflammatory cytokines (Figures 3I,J). Preliminary results indicate that IATL effectively inhibits the release of inflammatory factors, thereby contributing to the improvement of the inflammation-related microenvironment in pancreatic cancer.

### 3.4 NLRP3 is the direct target of IATL

**FIGURE 4:**
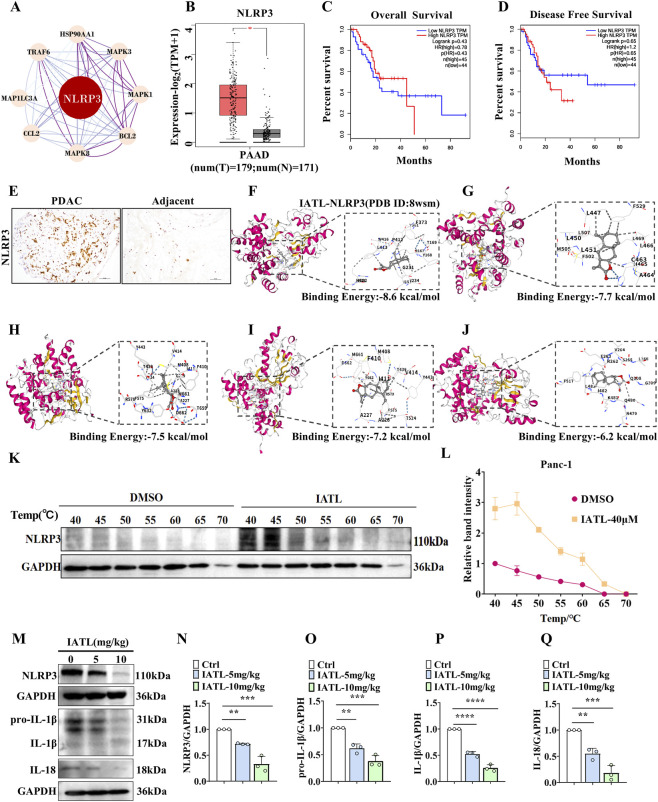
NLRP3 is a direct target of IATL. **(A)** Core PPI network of IATL targets in PDAC. **(B)** Expression levels of NLRP3 in PAAD (pancreatic adenocarcinoma) tissues and normal pancreatic tissues based on GEPIA database analysis. **(C,D)** Kaplan–Meier curves for overall survival and disease-free survival of pancreatic cancer patients with different NLRP3 expression levels. **(E)** Immunohistochemical analysis of NLRP3 in pancreatic cancer tissues. **(F–J)** Molecular docking simulation showing the interaction between the NLRP3 protein and the IATL molecule. **(K,L)** Cellular thermal shift assay (CETSA) analysis of drug–target protein interactions. Band intensities were quantified using ImageJ software. Data are presented as mean ± SD (n = 3). **(M–Q)** Western blot analysis of NLRP3, pro-IL-1β, IL-1β, and IL-18 protein levels in tumor specimens. Band intensities were quantified using ImageJ software. Data are presented as mean ± SD (n = 3). Statistical differences were determined by one-way ANOVA, where ns indicates no significant difference, *P < 0.05, **P < 0.01, ***P < 0.001, and ****P < 0.0001.

To identify potential antitumor molecular targets of IATL, a global network parameter analysis was performed on the intersecting genes using the Cytoscape plugin (Figure 4A). Particular attention was paid to NLRP3, which plays a critical role in inflammatory responses and tumor suppression. The expression levels of NLRP3 were compared between pancreatic cancer tissues and normal subjects using the GEPIA database (http://gepia.cancer-pku.cn). The results demonstrated that NLRP3 was overexpressed in PDAC tissues compared to normal pancreatic tissues (Figure 4B). Additionally, the database analyzed the overall survival (OS) and disease-free survival (DFS) of pancreatic cancer patients stratified by NLRP3-based biomarkers (Figures 4C,D). The results revealed statistically significant differences in OS and DFS between the two groups, indicating that NLRP3 is closely associated with the survival prognosis of pancreatic cancer patients. Further immunohistochemical (IHC) analysis of NLRP3 expression in pancreatic cancer tissues confirmed that NLRP3 expression was significantly increased in cancerous tissues compared to adjacent normal tissues (Figure 4E). Based on these findings, NLRP3 was hypothesized as a potential direct binding target of IATL in cancer cells. To validate this hypothesis, an interaction between IATL and NLRP3 was investigated. Molecular docking model predictions indicated that IATL could bind to the pocket of NLRP3 protein, with a minimum binding energy of −6.2 kcal/mol (Figures 4F–J). Subsequently, biophysical methods were employed to further validate the direct interaction between IATL and NLRP3 protein. CETSA demonstrated that IATL enhanced the thermal stability of NLRP3 (Figures 4K,L). Finally, WB was employed to detect the protein levels of NLRP3 and its downstream proteins IL-1β, pro-IL-1β, and IL-18 in mouse tumor tissues. The results demonstrated that IATL treatment significantly downregulated the expression of these proteins in a dose-dependent manner (Figures 4M–Q). Collectively, our findings indicate that NLRP3 is a potential target of IATL.

### 3.5 NLRP3 mediates IATL-induced regulation of inflammatory cytokine expression

**FIGURE 5:**
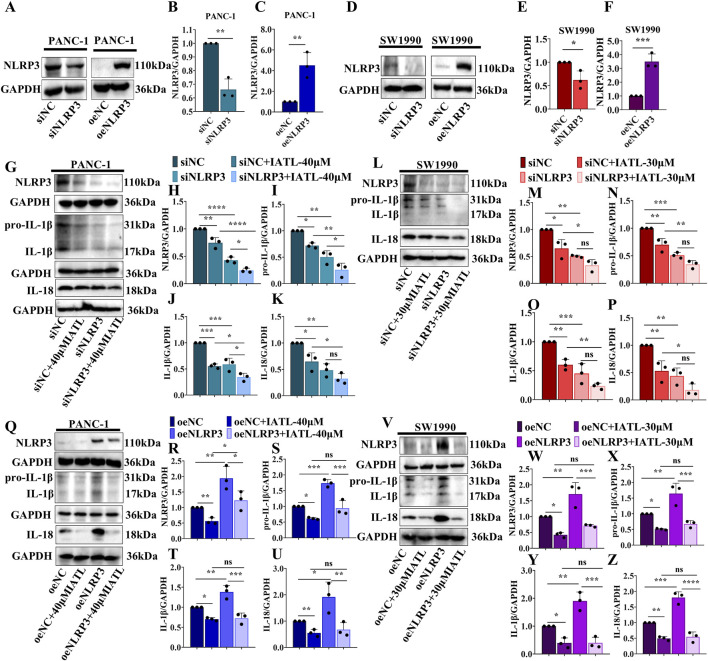
NLRP3 mediates IATL-induced regulation of inflammatory cytokine expression. **(A–F)** Western blot analysis of NLRP3 protein expression in PANC-1 and SW1990 cells after transfection with si-NLRP3 or NLRP3-overexpression plasmids. Band intensities were quantified using ImageJ software. Data are presented as mean ± SD (n = 3). **(G–Z)** Western blot analysis of NLRP3, pro-IL-1β, IL-1β, and IL-18 protein levels in pancreatic cancer cells following NLRP3 silencing or overexpression combined with IATL treatment. Band intensities were quantified using ImageJ software. Data are presented as mean ± SD (n = 3). Statistical differences were determined by one-way ANOVA, where ns indicates no significant difference, *P < 0.05, **P < 0.01, ***P < 0.001, and ****P < 0.0001.

It is known that the NLRP3/IL-1β pathway is a key inflammatory signal driving angiogenesis in the TEM. Therefore, IATL may inhibit pancreatic cancer angiogenesis by suppressing this critical inflammatory pathway. To further clarify the role of NLRP3 in IATL-mediated regulation of inflammatory cytokine expression, siRNA targeting NLRP3 (siNLRP3) and overexpression plasmids (oeNLRP3) were transfected into PANC-1 and SW1990 cells. WB was used to detect the protein level of NLRP3 in transfected cells. The results showed that silencing NLRP3 markedly decreased NLRP3 expression, whereas overexpression of NLRP3 significantly increased its protein level in both cell lines, indicating that the transfection efficiency was satisfactory (Figures 5A–F). Further analysis showed that in both PANC-1 and SW1990 cells, NLRP3 silencing combined with IATL treatment significantly reduced the expression levels of NLRP3, pro-IL-1β, IL-1β, and IL-18, whereas NLRP3 overexpression attenuated the inhibitory effect of IATL on these proteins (Figures 5G–Z). These findings suggest that NLRP3 mediates the inhibitory effect of IATL on inflammatory cytokine expression in pancreatic cancer cells.

### 3.6 NLRP3-mediated IATL regulates the angiogenic capacity of pancreatic cancer *in vitro*

**FIGURE 6:**
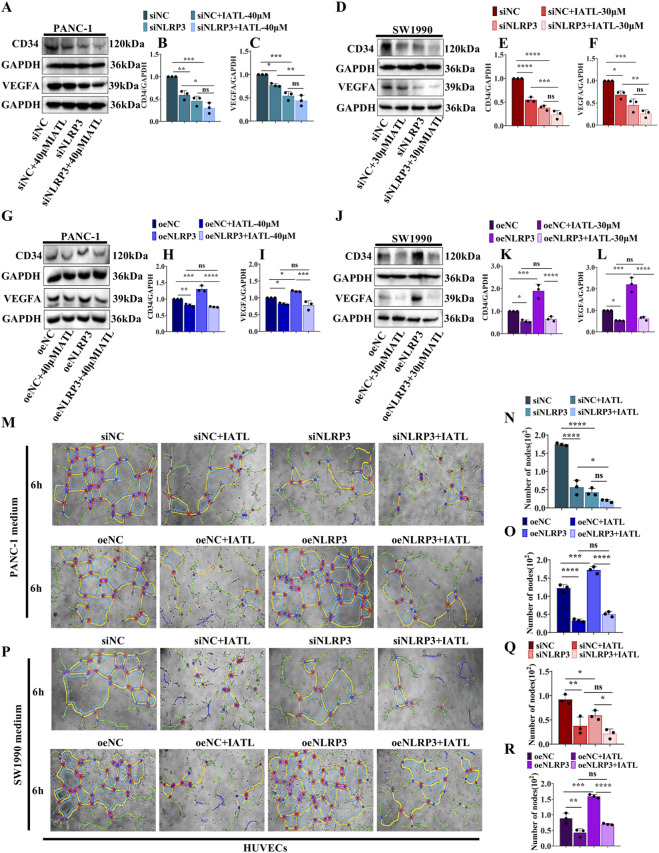
NLRP3-mediated IATL regulates the angiogenic capacity of pancreatic cancer *in vitro*. **(A–L)** Western blot analysis of VEGFA and CD34 protein expression in PANC-1 and SW1990 cells following NLRP3 silencing or overexpression combined with IATL treatment. Band intensities were quantified using ImageJ software. Data are presented as mean ± SD (n = 3). **(M–R)** Tube formation assays and quantification of total vascular branch points in HUVECs cultured with pancreatic cancer cell-conditioned medium following NLRP3 intervention combined with IATL treatment. Data are presented as mean ± SD (n = 3). Statistical differences were determined by one-way ANOVA, where ns indicates no significant difference, *P < 0.05, **P < 0.01, ***P < 0.001, and ****P < 0.0001.

To further investigate the regulatory role of NLRP3 in pancreatic cancer angiogenesis, the expression levels of angiogenesis-related proteins were detected by WB. The results showed that in both PANC-1 and SW1990 cells, silencing NLRP3 combined with IATL treatment significantly reduced the expression levels of angiogenesis marker proteins CD34 and VEGFA, whereas overexpression of NLRP3 reversed the inhibitory effect of IATL on these proteins (Figures 6A–L). To further evaluate the effect of pancreatic cancer cells on endothelial angiogenesis, conditioned medium was collected from the supernatants of different treatment groups and used to culture HUVECs *in vitro*. Tube formation assays showed that conditioned medium derived from PANC-1 and SW1990 cells treated with NLRP3 silencing combined with IATL markedly inhibited the tube formation ability of HUVECs, whereas overexpression of NLRP3 significantly reversed the suppressive effect of IATL on endothelial angiogenesis *in vitro* (Figures 6M–R). In addition, siNLRP3 and oeNLRP3 were transfected into HUVECs to further verify the direct role of NLRP3 in endothelial angiogenesis. WB and tube formation assays showed that NLRP3 silencing significantly reduced, whereas NLRP3 overexpression enhanced, the tube formation ability of HUVECs (Supplementary Figures S2A–H). These findings indicate that NLRP3 positively regulates the angiogenic process in pancreatic cancer, and that IATL inhibits pancreatic cancer angiogenesis by targeting NLRP3.

### 3.7 IATL inhibits tumor growth in orthotopic tumor-bearing mice by inhibiting NLRP3

**FIGURE 7:**
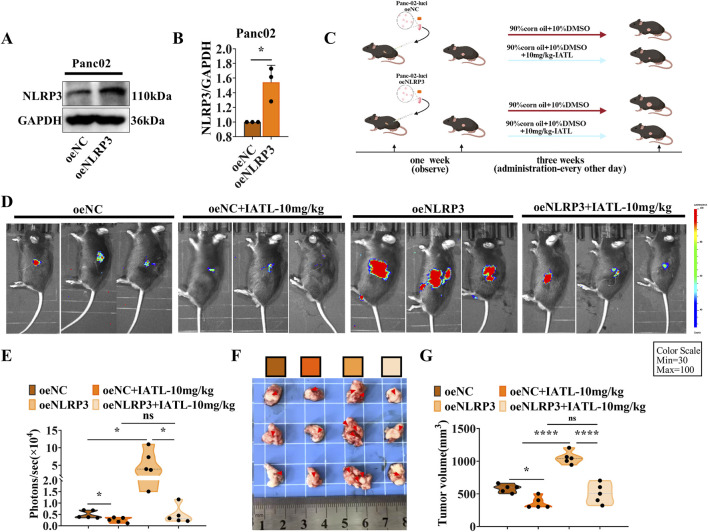
IATL delays tumor growth in orthotopic tumor-bearing mice by inhibiting NLRP3. Following NLRP3 overexpression, mice were intraperitoneally injected with vehicle or IATL (10 mg/kg) every other day for three consecutive weeks. **(A,B)** Western blot analysis confirming NLRP3 overexpression in Panc02 cells. Band intensities were quantified using ImageJ software. Data are presented as mean ± SD (n = 3). **(C)** Schematic illustration of the *in vivo* experimental design. **(D)** Analysis of the pancreatic cancer model using the *in vivo* imaging system (IVIS). **(E)** Quantification of tumor fluorescence signals in mice by IVIS. Data are presented as mean ± SD (n = 5). **(F,G)** Representative images and tumor volumes of mice. Data are presented as mean ± SD (n = 5). Statistical differences were determined by one-way ANOVA, where ns indicates no significant difference, *P < 0.05, **P < 0.01, ***P < 0.001, and ****P < 0.0001.

To elucidate the role of NLRP3 in tumor growth in orthotopic tumor-bearing mice, overexpression of NLRP3 was achieved in Panc02 cells through transfection (Figures 7A,B). Concurrently, an overexpressing NLRP3 Panc02 fluorescent cell line was established, and orthotopic injection was performed to establish a mouse pancreatic cancer model, followed by experimental interventions according to group assignments (Figure 7C). *In vivo* imaging in small animals demonstrated that the tumor region fluorescence signal was significantly enhanced in NLRP3-overexpressing tumor-bearing mice compared to the control group; however, this signal was markedly downregulated after IATL intervention (Figures 7D,E). Further tumor histological experiments showed high consistency with the *in vivo* imaging results (Figures 7F,G), providing histological validation of the aforementioned phenomena. In summary, NLRP3 promotes tumor growth in orthotopic pancreatic cancer mouse models by facilitating tumor cell proliferation, while IATL can inhibit this effect.

### 3.8 IATL inhibits angiogenesis in orthotopic tumor-bearing mice by suppressing NLRP3

**FIGURE 8:**
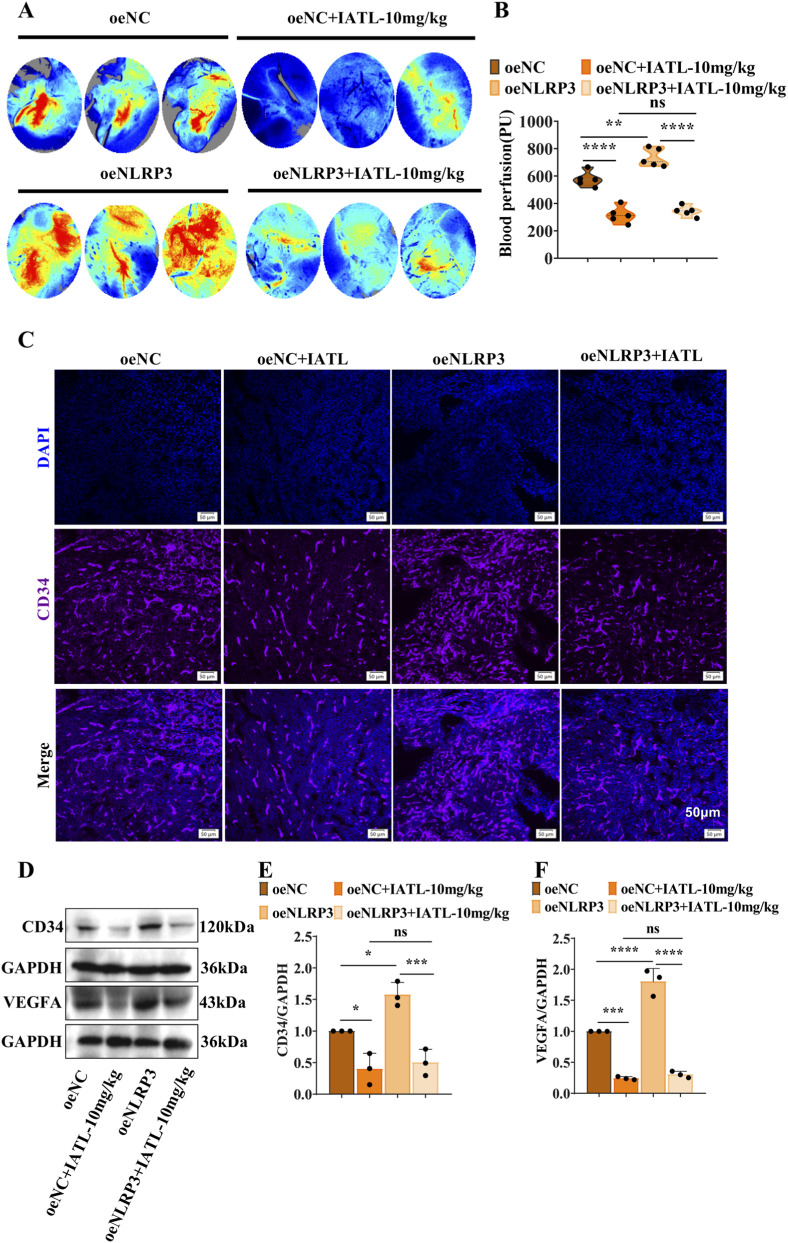
IATL inhibits angiogenesis in orthotopic tumor-bearing mice by suppressing NLRP3. **(A,B)** Laser speckle contrast imaging (LSCI) was used to evaluate blood perfusion in pancreatic cancer models. Data are presented as mean ± SD (n = 5). **(C)** Representative immunofluorescence images of CD34 (purple) staining in tumor tissues from each group. Nuclei were counterstained with DAPI (blue). Scale bar, 50 μm. **(D–F)** Western blot analysis evaluating the effects of *in vivo* NLRP3 overexpression on the expression of the angiogenesis markers CD34 and VEGFA. Band intensities were quantified using ImageJ software. Data are presented as mean ± SD (n = 3). Statistical differences were determined by one-way ANOVA, where ns indicates no significant difference, *P < 0.05, **P < 0.01, ***P < 0.001, and ****P < 0.0001.

Next, to clarify the effect of NLRP3 on tumor angiogenesis, laser speckle flow imaging technology was employed for detection (Figures 8A,B). Compared to the control group, the overexpression group exhibited higher tumor blood perfusion and greater vascular density, while IATL intervention reversed these phenomena. Concurrently, immunofluorescence staining of tumor tissues revealed that overexpression of NLRP3 increased the expression of angiogenesis marker protein CD34, which was reduced after IATL intervention, providing molecular-level evidence of its inhibitory effect (Figure 8C). To further validate the regulatory role of NLRP3 on tumor angiogenesis, WB assays were conducted to detect the expression levels of CD34 and VEGFA (Figures 8D–F). The results demonstrated that IATL treatment significantly inhibited the overexpression of CD34 and VEGFA induced by NLRP3 overexpression, further confirming that IATL exerts its anti-angiogenic effects by suppressing NLRP3-related pathways.

### 3.9 IATL alleviates tumor inflammatory microenvironment by inhibiting NLRP3

**FIGURE 9:**
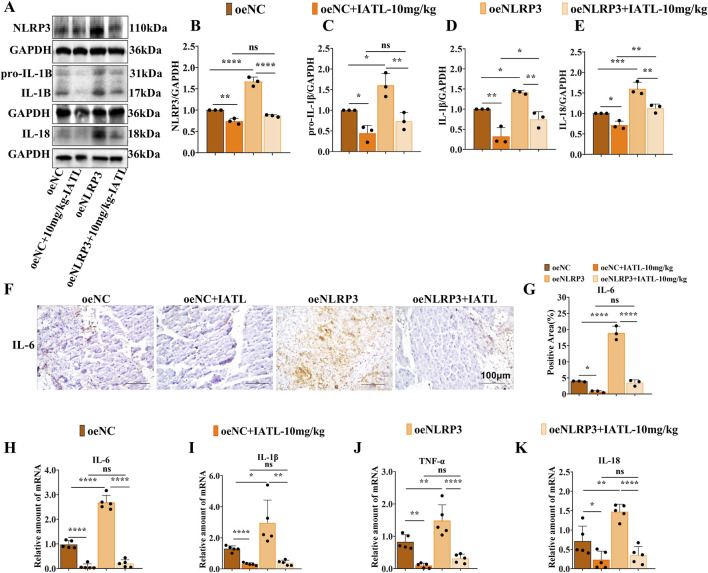
IATL alleviates the tumor inflammatory microenvironment by inhibiting NLRP3. **(A–E)** Western blot analysis of the effects of *in vivo* NLRP3 overexpression on pro-IL-1β, IL-1β, and IL-18 expression. Band intensities were quantified using ImageJ software. Data are presented as mean ± SD (n = 3). **(F,G)** Representative images of IL-6 immunohistochemical staining in tumor tissues from each group. Scale bar, 100 μm. Brown-stained areas were quantified using ImageJ software. **(H–K)** RT-qPCR analysis of mRNA levels of inflammatory factors IL-18, IL-1β, IL-6, and TNF-α in tumor specimens. Statistical differences were determined by one-way ANOVA, where ns indicates no significant difference, *P < 0.05, **P < 0.01, ***P < 0.001, and ****P < 0.0001.

The aforementioned study confirmed that IATL effectively inhibits tumor angiogenesis by suppressing NLRP3. It is known that the tumor inflammatory microenvironment is a key driver of pathological angiogenesis, and NLRP3 serves as a central hub in regulating this microenvironment. Therefore, we hypothesize that IATL’s inhibition of angiogenesis may be achieved by modulating the NLRP3-mediated inflammatory microenvironment. To validate the aforementioned hypothesis, the effects of IATL on the inflammatory microenvironment in an NLRP3 overexpression model were first analyzed at the protein level (Figures 9A–E). The results showed that overexpression of NLRP3 significantly upregulated the protein expression of pro-IL-1β, IL-1β, and IL-18 in tumor tissues, and IATL intervention effectively reversed this phenomenon. Additionally, immunohistochemistry (Figures 9F,G) and mRNA level assays further confirmed (Figures 9H–K) that NLRP3 overexpression significantly promotes the expression of multiple inflammatory factors, including IL-6, IL-1β, IL-18, and TNF-α, suggesting that it indirectly drives angiogenesis by activating the inflammatory microenvironment. In contrast, IATL treatment comprehensively downregulates the expression of these key inflammatory factors, with its effects highly correlated with the inhibition of the NLRP3 inflammatory pathway.

### 3.10 IATL inhibits the activation of NLRP3 inflammasomes

**FIGURE 10:**
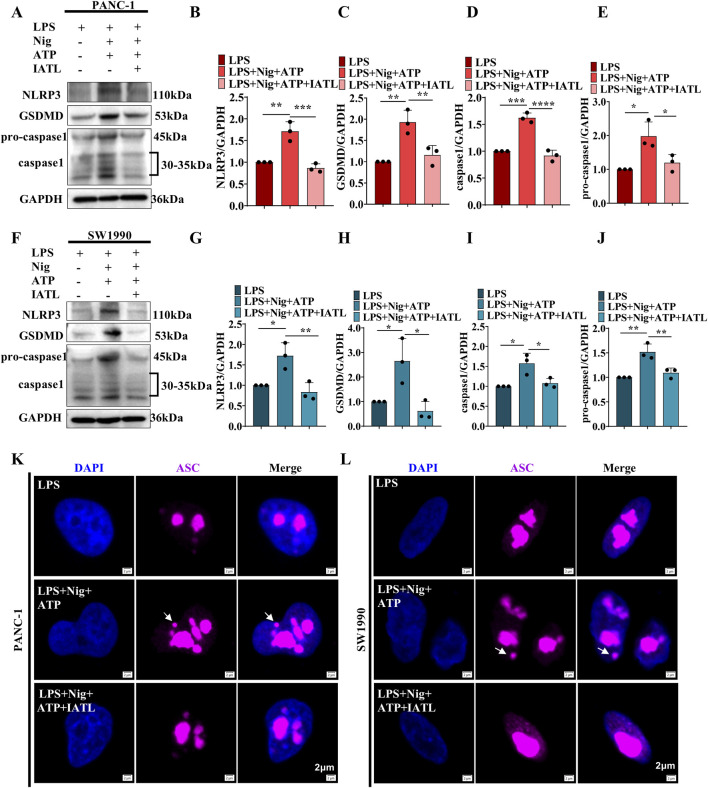
IATL inhibits the activation of NLRP3 inflammasomes. **(A–J)** Western blot analysis of GSDMD, pro-caspase-1, and caspase-1 protein levels in PANC-1 and SW1990 cells under LPS co-stimulation. Band intensities were quantified using ImageJ software. Data are presented as mean ± SD (n = 3). **(K,L)** Formation of ASC (purple) specks in PANC-1 and SW1990 cells following IATL treatment. Scale bar, 2 μm (n = 3). Statistical differences were determined by one-way ANOVA, where ns indicates no significant difference, *P < 0.05, **P < 0.01, ***P < 0.001, and ****P < 0.0001.

To further clarify whether IATL regulates the activation of NLRP3 inflammasomes, an NLRP3 inflammasome activation model induced by LPS combined with Nigericin/ATP was established in PANC-1 and SW1990 cells. As shown in Figures 10A–J, compared with the LPS group, the expression levels of NLRP3, GSDMD, caspase-1, and pro-caspase-1 were significantly increased after stimulation with LPS combined with Nigericin/ATP in both PANC-1 and SW1990 cells, indicating successful activation of the NLRP3 inflammasome. After IATL treatment, the expression levels of these proteins were markedly decreased in both cell lines (Figures 10A–J), suggesting that IATL effectively inhibited NLRP3 inflammasome activation.

To further verify the inhibitory effect of IATL on inflammasome assembly, an ASC fluorescent speck assay was performed. In THP-1 cells, ASC aggregation induced by NLRP3 inflammasome activation appeared as typical round and bright fluorescent specks, confirming that the ASC speck assay could effectively detect inflammasome assembly (Supplementary Figure S3). Consistently, obvious ASC speck formation was observed in PANC-1 and SW1990 cells after stimulation with LPS combined with Nigericin/ATP, whereas IATL treatment significantly reduced ASC speck formation (Figures 10K,L), indicating that IATL suppressed the assembly of NLRP3 inflammasomes. These findings suggest that IATL inhibits both the activation and assembly of NLRP3 inflammasomes.

## 4 Discussion

As our understanding of the molecular mechanisms underlying tumorigenesis and progression deepens, research has increasingly focused on the discovery of effective anti-cancer components from natural products and elucidating their mechanisms of action. In fact, many conventional chemotherapeutic agents, including paclitaxel, etoposide, and vinca alkaloids, are plant-derived compounds (Asma et al., 2022). IATL is a terpenoid compound extracted from the roots of traditional Chinese medicinal herbs. It exhibits diverse pharmacological activities, including anti-inflammatory and immunomodulatory effects, and has demonstrated therapeutic potential for various inflammatory diseases, such as obesity (Huang et al., 2023; Yang et al., 2024; Jung et al., 2022). In addition, some studies have revealed that IATL displays anti-tumor activity in multiple cancer types, including hepatocellular carcinoma, ovarian cancer, and triple-negative breast cancer, in both *in vitro* and *in vivo* models (Wang et al., 2016; Kim et al., 2021; Chun, 2023). These findings highlight IATL as a promising candidate for cancer therapy. However, the anti-tumor effects of IATL in PDAC and its underlying mechanisms remain unclear. Our study demonstrates that IATL significantly inhibits cell proliferation and migration *in vitro*, and that it markedly suppresses tumor growth and angiogenesis *in vivo*. Additionally, bioinformatics analysis revealed that IATL-associated genes were enriched in multiple inflammatory signaling pathways, including NF-κB and JAK/STAT3 signaling, suggesting that IATL may exert anti-PDAC effects, at least in part, by modulating the TME.

The TME is a complex system composed of cancer cells, immune cells, stromal cells, and the extracellular matrix (ECM). Chronic inflammation not only shapes the immunosuppressive TME but is also a key driver in accelerating tumor proliferation, invasion, and immune evasion. These processes involve dynamic interactions among multiple cells and factors, forming a vicious cycle of tumorigenesis, making inflammation a critical therapeutic target in cancer (Leuti et al., 2020). Inflammatory cells secrete cytokines and chemokines, which, together with the ECM, form a distinct microenvironment, known as the inflammatory TME, which promotes continuous renewal and proliferation of tumor cells, driving tumor progression and metastasis. For example, Li et al. demonstrated that cancer cell-derived CXCL1, a key inflammatory chemokine, contributes to T-cell exclusion and immunotherapy resistance in PDAC (Li et al., 2018). Similarly, Ganguly K et al. observed that pancreatic fibroblasts release growth factors, chemokines, and ECM components, thereby sustaining a malignant cycle of fibro-inflammatory stroma development (Ganguly et al., 2022). Furthermore, our previous studies demonstrated that inflammatory chemokines such as CCL20 and CXCL5 are overexpressed in pancreatic cancer (Chen et al., 2021). The interplay between inflammation and the TME serves as an “engine” for cancer progression, and targeting this axis may represent a novel strategy to overcome current therapeutic limitations.

In this study, through *in vitro* and *in vivo* experiments, we found that IATL significantly reduces the secretion of inflammatory cytokines, including IL-6, IL-18, TNF-α, and IL-1β, in pancreatic cancer cells. These cytokines are known to activate STAT3 and/or NF-κB in an autocrine or paracrine manner, promoting cancer cell survival, proliferation, and the maintenance of a pro-tumorigenic inflammatory TME, thereby exacerbating PDAC progression (Gukovsky et al., 2013). Furthermore, IL-6 and TNF-α exhibit oncogenic functions in PDAC (Chen et al., 2025; Zhang et al., 2024), and elevated IL-6 correlates with advanced tumor stage and poor survival (Yako et al., 2016), while TNF-α facilitates metastasis by inducing angiogenesis (Padoan et al., 2019). As such, suppressing the secretion of inflammatory cytokines in pancreatic cancer has significant therapeutic value, as this strategy could directly inhibit tumor growth, enhance chemotherapy efficacy, block the formation of an inflammatory TME, and modulate anti-tumor immune responses (Roshani et al., 2014). Together, the findings from this study suggest that IATL exerts its anti-PDAC effects primarily through its potent anti-inflammatory properties.

To further elucidate the core mechanism of action of IATL, we identified that IATL can specifically bind to NLRP3 protein through network pharmacology-based screening of key target genes. This finding aligns with previous reports demonstrating that IATL and its derivatives target the NLRP3 inflammasome to ameliorate inflammatory diseases such as colitis (Zhao et al., 2024).

NLRP3 is a crucial inflammasome sensor protein that plays a central role in regulating innate immune responses and inflammatory signaling pathways. It is involved in numerous biological processes, including immune function, cell growth, differentiation, and hematopoiesis, and has been implicated in cancer initiation, progression, metastasis, survival, and therapeutic resistance in human malignancies (Ma, 2023). In addition, increasing evidence indicates that NLRP3-mediated inflammation contributes to the pathogenesis of PDAC, with activation of the NLRP3 pathway promoting tumor growth and chemoresistance (Yang et al., 2023; Yang et al., 2025; Zheng and Liu, 2022). Moreover, clinical studies have identified the aberrant activation of the NLRP3 inflammasome in pancreatic cancer tissues, which induces the release of pro-inflammatory cytokines such as IL-1β and IL-18, thereby enhancing tumor proliferation, metastasis, and resistance to chemotherapy (Németh et al., 2025; Amo-Aparicio et al., 2023). Notably, inhibition of NLRP3 inflammasome activation has been shown to suppress pancreatic cancer viability, growth, and metastasis (Gu et al., 2022). In addition, Zhang et al. demonstrated that flavonoids inhibit NLRP3 inflammasome activation to alleviate pancreatic fibrosis in chronic pancreatitis (Zhang G. et al., 2021), while Yang et al. reported that cordycepin protects against acute pancreatitis and pancreatic acinar cell damage through AMPK-mediated regulation of NF-κB and NLRP3 inflammasome activation (Yang et al., 2020). In addition, GALNT6 knockdown was found to promote PDAC cell pyroptosis through NF-κB/NLRP3/GSDMD and GSDME signaling (Liu et al., 2020). Importantly, NLRP3-associated inflammatory mediators have been implicated in promoting tumor progression and metastasis by modulating angiogenesis (Huang et al., 2024; Shi et al., 2023), and CpG-B oligonucleotides counteracts immunosuppression by inhibiting NLRP3 pathway activation (Zhao et al., 2021).

In the present study, IATL significantly suppressed NLRP3 expression, suggesting this is a mechanism for its anti-PDAC effects. Notably, our results further showed that IATL reduced the expression of GSDMD, caspase-1, and pro-caspase-1 in LPS plus nigericin/ATP-stimulated pancreatic cancer cells, and markedly decreased ASC speck formation, indicating that IATL inhibits not only NLRP3 expression but also inflammasome activation and assembly. These findings extend previous observations on the anti-inflammatory activity of IATL and provide additional mechanistic evidence that IATL disrupts the NLRP3/IL-1β axis at the inflammasome level. However, the precise mechanisms by which the NLRP3 contributes to PDAC development remain to be fully investigated, motivating continued exploration of how IATL exerts its anti-PDAC effects through modulation of NLRP3-mediated inflammatory pathways.

Tumor angiogenesis is crucial for tumor growth and metabolism, and it is a complex process regulated by a balance of pro-angiogenic and anti-angiogenic factors within solid tumors (Bergers and Benjamin, 2003). When pro-angiogenic factors dominate, endothelial cells are stimulated to proliferate and migrate toward the tumor, promoting the formation of new blood vessels. However, excessive levels of pro-angiogenic factors can further stimulate abnormal angiogenesis (Martin et al., 2019). In addition, the structural and functional disorders of these abnormal blood vessels lead to chaotic blood flow, leading to a heterogeneous TME. The PDAC TME exhibits typical spatial vascular heterogeneity: low vascular permeability within the tumor core, while the peripheral tumor margins demonstrate abundant vasculature and high perfusion. This model accurately replicates these physiological characteristics. In this environment, tumor cells are more likely to invade and infiltrate surrounding tissues, ultimately leading to irreversible tumor malignancy.

Some studies have demonstrated that the inflammatory microenvironment in pancreatic cancer significantly promotes angiogenesis, which is closely associated with poor patient prognosis (Padoan et al., 2019; Farajzadeh Valilou et al., 2018). For example, Li et al. found that tumor cells secrete VEGF and hypoxia-inducible factor (HIF) to promote angiogenesis and exacerbate tumor progression (Li et al., 2019). We conducted a co-expression analysis of IATL and PAAD targets using an online database, identified the overlapping genes through VENNY screening, and performed GO enrichment analysis on these genes. The results revealed that IATL-regulated genes were significantly enriched in angiogenesis-related biological processes, suggesting that IATL likely plays a crucial role in regulating angiogenesis. Consequently, we investigated the effects of IATL on angiogenesis in both *in vivo* and *in vitro* PDAC models, demonstrating its significant inhibitory effect on PDAC angiogenesis. To elucidate the downstream angiogenesis signaling pathways regulated by NLRP3, we examined two key angiogenesis proteins, VEGFA and CD34. For example, previous studies have shown that VEGF is regulated by IL-6 in pancreatic cancer cells, where excessive VEGF stimulates angiogenesis and tumor vascular formation (Roshani et al., 2014). Likewise, CD34 is regulated by BICC1 and promotes angiogenesis-driven PDAC progression (Huang et al., 2023). Our results demonstrate that IATL inhibits the expression of VEGFA and CD34 both *in vitro* and *in vivo*, whereas this inhibitory effect is attenuated by NLRP3 overexpression. Consistently, conditioned medium from IATL-treated pancreatic cancer cells markedly impaired HUVECs tube formation, while restoration of NLRP3 partially reversed this effect. Together, these findings support the notion that NLRP3 acts as an important molecular bridge linking inflammatory signaling to angiogenesis in PDAC, and that IATL suppresses malignant progression, at least in part, by disrupting this inflammation-angiogenesis axis.

## 5 Conclusion

In conclusion, our findings demonstrate that IATL significantly inhibits the proliferation and migration of PDAC cells and interferes with angiogenesis, potentially by suppressing inflammation. Further network pharmacology analysis and experimental validation demonstrated that IATL targets the NLRP3 signaling pathway, suppresses NLRP3 inflammasome activation, and exerts both anti-inflammatory and anti-angiogenic effects (Graphical abstract). Together, these results suggest that IATL is a potential candidate for the treatment of PDAC.

## Data Availability

The raw data supporting the conclusions of this article will be made available by the authors, without undue reservation.

## Supplementary material

SUPPLEMENTARY FIGURE S1 IATL exhibits significant anticancer activity *in vitro* against pancreatic cancer. **(A)** Effects of IATL on the viability of PANC-1, Panc02, and SW1990 cells, as determined by MTT assay. Data are presented as mean ± SD (n = 5). **(B)** Continuous kinetic monitoring of the viability of PANC-1, Panc02, and SW1990 cells over 72 h following IATL treatment. Images were captured using the Cytation 7 live-cell imaging system. **(C)** Morphological changes in PANC-1, Panc02, and SW1990 cells after treatment with IATL. Images were captured using the Cytation 7 live-cell imaging system. **(D–F)** Representative images from wound-healing assays for Panc02 and SW1990 cells incubated with 0, 5, 10, or 20 μM IATL, and for PANC-1 cells incubated with 0, 10, 20, or 40 μM IATL. Images were captured using the Cytation 7 live-cell imaging system. **(G–I)** Quantification of wound area using ImageJ software. The migration rate was expressed as a percentage of the control. Data are presented as mean ± SD (n = 3). Statistical differences were determined by one-way ANOVA, where ns indicates no significant difference, *P < 0.05, **P < 0.01, ***P < 0.001, and ****P < 0.0001.

SUPPLEMENTARY FIGURE S2 The effect of NLRP3 expression changes on endothelial cell tube formation. **(A–D)** Western blot analysis of protein expression levels in HUVECs following NLRP3 silencing and overexpression. Band intensities were quantified using ImageJ software. Data are presented as mean ± SD (n = 3). **(E–H)** Effects of NLRP3 silencing and overexpression on tube formation in HUVECs. Data are presented as mean ± SD (n = 3). Statistical differences were determined by one-way ANOVA, where ns indicates no significant difference, *P < 0.05, **P < 0.01, ***P < 0.001, and ****P < 0.0001.

SUPPLEMENTARY FIGURE S3 The ASC fluorescence speck assay detects the assembly of NLRP3 inflammasomes. Morphology of fluorescent specks formed by ASC aggregation following NLRP3 activation in THP-1 cells.
