# Supplementary figures and images for: Isoalantolactone inhibits pancreatic ductal adenocarcinoma progression via direct targeting of NLRP3-mediated inflammation-angiogenesis axis
Source: Front Pharmacol. 2026 Jul 15;17:1840118. doi: 10.3389/fphar.2026.1840118 (PMC13414130; doi:10.3389/fphar.2026.1840118)

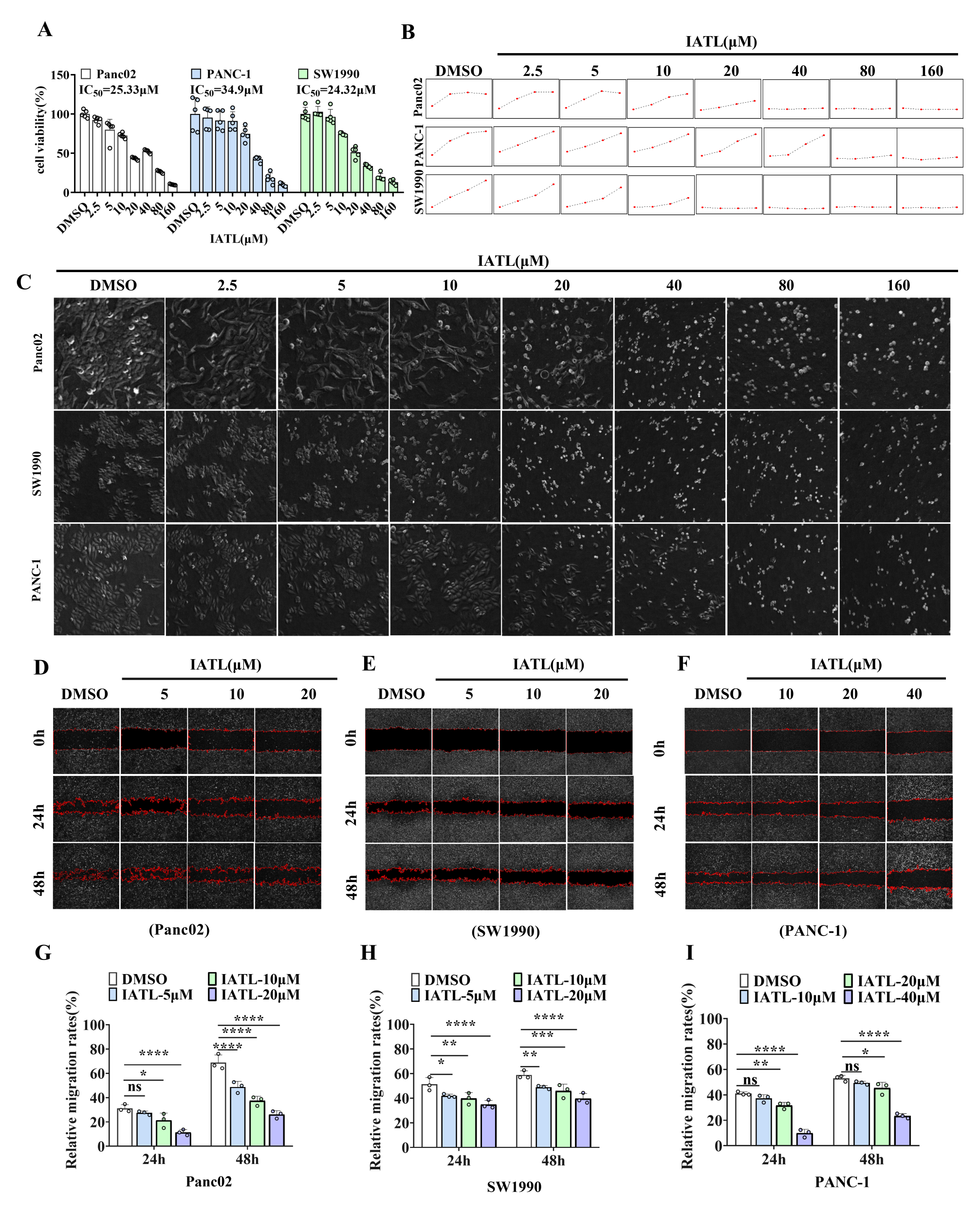

Supplement: Supplementary file 1 [file Image1.tiff]

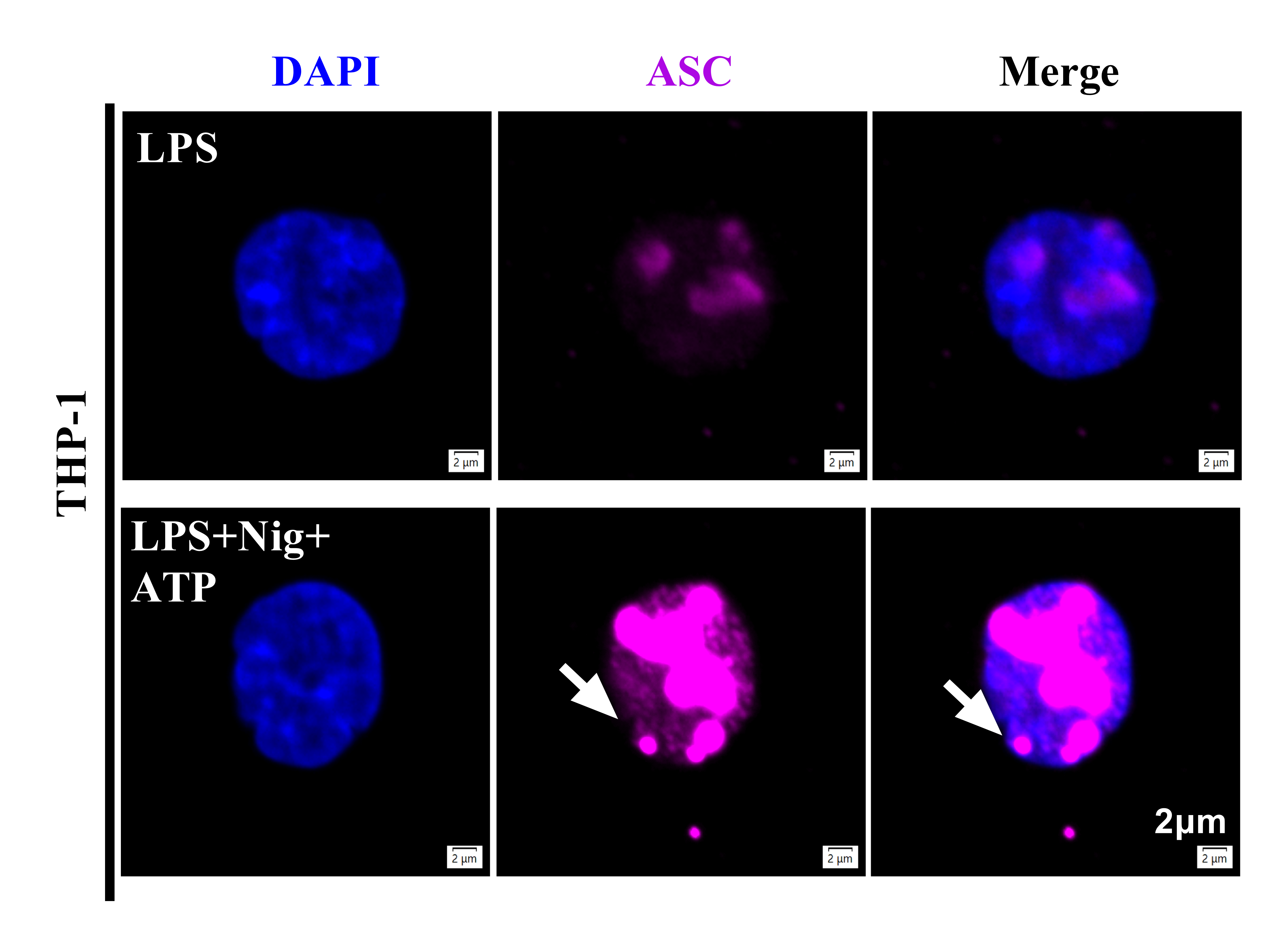

Supplement: Supplementary file 2 [file Image3.tif]
