# Supplementary material for: Isoalantolactone inhibits pancreatic ductal adenocarcinoma progression via direct targeting of NLRP3-mediated inflammation-angiogenesis axis
Source: Front Pharmacol. 2026 Jul 15;17:1840118. doi: 10.3389/fphar.2026.1840118 (PMC13414130; doi:10.3389/fphar.2026.1840118)
Supplement: Supplementary file 4 [file DataSheet1.pdf]

Supplementary Table 1. The information of western blot antibodies

Name	Host	Dilution	Catalog	Socure
Anti-NLRP3	Rabbit	1:1000	DF7438	Affinity
Anti-IL-1B	Rabbit	1:2000	A16288	ABclonal
Anti-VEGFA	Rabbit	1:2000	A0280	ABclonal
Anti-CD34	Rabbit	1:2000	A19015	ABclonal
Anti-PCNA	Rabbit	1:2000	A12427	ABclonal
Anti-GAPDH	Rabbit	1:10000	20536	Proteintech
Anti-Caspase1	Rabbit	1:2000	81482-1-RR	Proteintech
Anti-GSDMD	Rabbit	1:2000	20770-1-AP	Proteintech
Anti-IL-18	Rabbit	1:2000	A1115	ABclonal
Anti-ASC/TMS1	Rabbit	1:2000	A1170SP	ABclonal
HRP-Anti-Mouse		1:10000	AS003	ABclonal
HRP-Anti-Rabbit		1:10000	AS014	ABclonal

Supplementary Table 2. Primer sequences for RT-qPCR.

RNA name	FORWARD	REVERSE
18S	GAAGGGCACCACCAGGAGT	CAGACAAATCACTCCACCAA
TNF-α	GCCACCACGCTCTTCTGTCTAC	GGTTTGTGAGTGTGAGGGTCTGG
IL-6	TGGGACTGATGCTGGTGACA	ACAGGTCTGTTGGGAGTGGT
IL-1β	TGAAGGGCTGCTTCCAAACC	GCCTGCCTGAAGCTCTTGTT
IL-18	TCAAAGTGCCAGTGAACCCC	GGTCACAGCCAGTCCTCTTAC

Supplementary Table 3. The siRNA sequences

siRNA name	Sense	Antisense
si-NC	UUCUCCGAACGUGUCACGUTT	ACGUGACACGUUCGGAGAATT
SiNLRP3	GGAUCUUUGCAGCGAUCAATT	UUGAUCGCUGCAAAGAUCCTT

NOTE: Please save this file locally before filling in the table, DO NOT work on the file within your internet browser as changes will not be saved. Adobe Acrobat Reader (available free here) is recommended for completion.

The ARRIVE guidelines 2.0: author checklist

The ARRIVE Essential 10

These items are the basic minimum to include in a manuscript. Without this information, readers and reviewers cannot assess the reliability of the findings.

Item		Recommendation	Section/line number, or reason for not reporting
**Study design**	1	For each experiment, provide brief details of study design including:	
		a. The groups being compared, including control groups. If no control group has been used, the rationale should be stated.	Control group (Vehicle), Low dose IATL group (5 mg/kg), High dose IATL group (10 mg/kg). For mechanism validation: NLRP3 Overexpression (OE) group vs. NLRP3 OE + IATL (10 mg/kg) group.
		b. The experimental unit (e.g. a single animal, litter, or cage of animals).	Single animal (individual C57BL/6 mouse).
**Sample size**	2	a. Specify the exact number of experimental units allocated to each group, and the total number in each experiment. Also indicate the total number of animals used.	Total used: 60 mice. Allocation: Initially 7 9 mice were inoculated per group to ensure sufficient tumor bearing animals. Final n: The final sample size for analysis was [illegible]
		b. Explain how the sample size was decided. Provide details of any *a priori* sample size calculation, if done.	Sample size was determined based on preliminary experiments to ensure sufficient statistical power to detect significant differences in tumor volume.
**Inclusion and exclusion criteria**	3	a. Describe any criteria used for including and excluding animals (or experimental units) during the experiment, and data points during the analysis. Specify if these criteria were established *a priori*. If no criteria were set, state this explicitly.	Mice with detectable bioluminescence signals (IVIS) above background levels one week post inoculation were included.
		b. For each experimental group, report any animals, experimental units or data points not included in the analysis and explain why. If there were no exclusions, state so.	Animals that failed to develop tumors or died of surgical complications before treatment initiation were excluded. No animals were excluded after treatment randomization.
		c. For each analysis, report the exact value of *n* in each experimental group.	Analysis was performed on n=5 mice per group.
**Randomisation**	4	a. State whether randomisation was used to allocate experimental units to control and treatment groups. If done, provide the method used to generate the randomisation sequence.	Mice were randomly assigned to treatment or control groups using a random number generator after tumor establishment and prior to the first treatment.
		b. Describe the strategy used to minimise potential confounders such as the order of treatments and measurements, or animal/cage location. If confounders were not controlled, state this explicitly.	Cage location was rotated.
**Blinding**	5	Describe who was aware of the group allocation at the different stages of the experiment (during the allocation, the conduct of the experiment, the outcome assessment, and the data analysis).	The investigators performing the animal treatments were aware of the group allocation due to the distinct appearance of the drug solution. However, outcome assessments (including tumor volume measurement, quantification of IVIS/LSCI signals, and histological scoring) were performed in a blinded manner. Samples and image files [illegible]
**Outcome measures**	6	a. Clearly define all outcome measures assessed (e.g. cell death, molecular markers, or behavioural changes).	Tumor growth (measured by IVIS fluorescence signal intensity and tumor volume at necropsy) and Angiogenesis (measured by blood perfusion via Laser Speckle Contrast Imaging/LSCI).
		b. For hypothesis-testing studies, specify the primary outcome measure, i.e. the outcome measure that was used to determine the sample size.	
**Statistical methods**	7	a. Provide details of the statistical methods used for each analysis, including software used.	Data were analyzed using One way ANOVA for multiple group comparisons. Statistical analysis was performed using GraphPad Prism 8.0.1 software.
		b. Describe any methods used to assess whether the data met the assumptions of the statistical approach, and what was done if the assumptions were not met.	Data distribution was assumed to be normal; data are presented as Mean ± SD.
**Experimental animals**	8	a. Provide species-appropriate details of the animals used, including species, strain and substrain, sex, age or developmental stage, and, if relevant, weight.	18 20g C57BL/6 male mice
		b. Provide further relevant information on the provenance of animals, health/immune status, genetic modification status, genotype, and any previous procedures.	purchased from the Animal Center of Guizhou Medical University
**Experimental procedures**	9	For each experimental group, including controls, describe the procedures in enough detail to allow others to replicate them, including:	
		a. What was done, how it was done and what was used.	Model: Orthotopic pancreatic cancer model established by injecting Panc02 luci cells into the pancreas.
		b. When and how often.	Intervention: Intraperitoneal (i.p.) injection of IATL or Vehicle.
		c. Where (including detail of any acclimatisation periods).	Dosing: 5 mg/kg or 10 mg/kg, administered every other day. Duration: Treatment continued for 3 consecutive weeks.
		d. Why (provide rationale for procedures).	To evaluate the anti tumor and anti angiogenic efficacy of IATL in a clinically relevant microenvironment.
**Results**	10	For each experiment conducted, including independent replications, report:	
		a. Summary/descriptive statistics for each experimental group, with a measure of variability where applicable (e.g. mean and SD, or median and range).	Data are reported as Mean±SD for each group (n=5).
		b. If applicable, the effect size with a confidence interval.	Not applicable
